# *Gardnerella vaginalis* clades in pregnancy: New insights into the interactions with the vaginal microbiome

**DOI:** 10.1371/journal.pone.0269590

**Published:** 2022-06-14

**Authors:** Marco Severgnini, Sara Morselli, Tania Camboni, Camilla Ceccarani, Melissa Salvo, Sara Zagonari, Giulia Patuelli, Maria Federica Pedna, Vittorio Sambri, Claudio Foschi, Clarissa Consolandi, Antonella Marangoni

**Affiliations:** 1 Institute of Biomedical Technologies – National Research Council, Segrate, Milan, Italy; 2 Microbiology, DIMES, University of Bologna, Bologna, Italy; 3 Family Advisory Health Centres, Ravenna, Italy; 4 Unit of Microbiology, Greater Romagna Hub Laboratory, Pievesestina di Cesena, Italy; GGD Amsterdam, NETHERLANDS

## Abstract

*Gardnerella vaginalis* (GV) is an anaerobic bacterial species involved in the pathogenesis of bacterial vaginosis (BV), a condition of vaginal dysbiosis associated with adverse pregnancy outcomes. GV strains are categorized into four clades, characterized by a different ability to produce virulence factors, such as sialidase. We investigated the distribution of GV clades and sialidase genes in the vaginal ecosystem of a cohort of pregnant women, assessing the correlations between GV clades and the whole vaginal microbiome. A total of 61 Caucasian pregnant women were enrolled. Their vaginal swabs, collected both at the first and third trimester of pregnancy, were used for (i) evaluation of the vaginal status by Nugent score, (ii) vaginal microbiome profiling by 16S rRNA sequencing, (iii) detection and quantification of GV clades and sialidase A gene by qPCR assays. DNA of at least one GV clade was detected in most vaginal swabs, with clade 4 being the most common one. GV clade 2, together with the presence of multiple clades (>2 simultaneously), were significantly associated with a BV condition. Significantly higher GV loads and sialidase gene levels were found in BV cases, compared to the healthy status. Clade 2 was related to the major shifts in the vaginal microbial composition, with a decrease in *Lactobacillus* and an increase in several BV-related taxa. As the number of GV clades detected simultaneously increased, a group of BV-associated bacteria tended to increase as well, while *Bifidobacterium* tended to decrease. A negative correlation between sialidase gene levels and *Lactobacillus*, and a positive correlation with *Gardnerella*, *Atopobium*, *Prevotella*, *Megasphaera*, and *Sneathia* were observed. Our results added knowledge about the interactions of GV clades with the inhabitants of the vaginal microbiome, possibly helping to predict the severity of BV and opening new perspectives for the prevention of pregnancy-related complications.

## Introduction

In healthy reproductive-aged women, the vaginal microbiome is characterized by a low bacterial diversity, often dominated by different species of the *Lactobacillus* genus [1–3]. However, when a deep change in bacterial profiles occurs, a dysbiosis condition called bacterial vaginosis (BV) may arise. BV is characterized by a depletion of lactobacilli, combined with the growth of a polymicrobial community of anaerobic species [4]. Among these species, *Gardnerella vaginalis* (GV) is thought to play a key role in the pathogenesis of BV [5]. Indeed, GV can produce various virulence factors—including sialidase, a hydrolase able to degrade local immunoglobulin A (IgA) and vaginal mucins, likely contributing to the diminished viscosity of local secretion, resulting in an increased vulnerability to pathogens [6,7]. Moreover, GV has a great ability to form a biofilm acting as a scaffold to which other anaerobic species, such as *Atopobium vaginae* and *Prevotella* spp., can subsequently attach [8,9].

Recent genomic analyses based on the sequencing of a chaperonin-60 gene region (*cpn60*) have revealed that the GV population structure consists of four clades. Each clade represents a distinct gene pool with specific genomic properties [10].

It is not clear yet whether the presence and the distribution of the different GV clades in the vaginal environment are universally applicable fingerprints of vaginal health or disease. Previous studies have reported contrasting results about the association between GV clades and the BV condition, although it is known that all four clades are present at higher abundances in women with abnormal vaginal flora [11–14].

At present, little is known about the distribution of the different GV clades in the vaginal environment of pregnant women. Considering that increased levels of BV-associated bacteria in the vaginal microbiome can negatively affect both maternal and neonatal health, the assessment of GV clade distribution during pregnancy can deepen the pathogenesis of dysbiotic conditions and open the way to new strategies for the prevention of obstetric complications (e.g., preterm birth) [15–17].

Therefore, the aim of this study was to investigate the prevalence and distribution of GV clades in the vaginal ecosystem of pregnant women at two different gestational stages (first and third trimester). Moreover, to shed light on the pathobiology of GV clades, we assessed the (i) correlation between the different clades and the vaginal microbiome profiles (by means of 16S rRNA), and (ii) the presence and quantification of the sialidase A gene in the vaginal environment.

## Materials and methods

### Study group and sample collection

From April 2019, a total of 61 Caucasian pregnant women attending Family Advisory Health Centers of Ravenna (Italy) for prenatal care were enrolled for the study.

The exclusion criteria were: (i) age <18 years; (ii) HIV positivity; (iii) body mass index (BMI) > 33; (iv) medically assisted procreation; (v) use of any antibiotics in the month preceding the examination; (vi) use of vaginal douches or topical agents in the last two weeks; (vii) presence of uncontrolled chronic diseases (e.g., diabetes, autoimmune disorders, malignancies); (viii) drug addiction or heavy smokers (>15 cigarettes/day). Moreover, women with urogenital infections due to sexually transmitted pathogens (*Chlamydia trachomatis*, *Neisseria gonorrhoeae*, *Trichomonas vaginalis*, *Mycoplasma genitalium*), to aerobic vaginitis, or to symptomatic candidiasis were excluded after the laboratory testing.

A vaginal swab (E-swab, Copan, Brescia, Italy) from each woman was collected at gestational stages between 9–12 weeks (first trimester) and between 31–34 weeks (third trimester).

For all patients, demographic data and information about urogenital symptoms were recorded. A written informed consent was obtained from all subjects and the study protocol was approved by the Ethics Committee of Romagna (CEROM) (n° 2032 of 21^st^ February 2018). This study was carried out in accordance with the Declaration of Helsinki, following the recommendations of the Ethics Committee.

### Microbiological investigations

Pathogens responsible for sexually transmitted infections were excluded by a commercial nucleic acid amplification technique (NAAT, Seeplex STI Master Panel 1; Seegene, Seoul, KR). Microscopic examination and semi-quantitative cultures were performed for aerobic vaginitis and candidiasis diagnosis [18,19].

The composition of the vaginal microbiota was evaluated by a Gram stain scoring system (Nugent score). Based on this score, women were divided into three groups: “H” (score 0–3; normal lactobacilli-dominated microbiota), “I” (score 4–6; intermediate microbiota), “BV” (score 7–10; bacterial vaginosis) [20].

### Vaginal microbiome profiling

Nucleic acids were extracted from vaginal swabs by means of the Versant molecular system (Siemens Healthcare Diagnostics, Tarrytown, NY, USA) equipped with a sample preparation module designed for automated sample preparation [21]. As previously reported, Versant system performs a highly efficient DNA extraction and purification by means of proteinase K, a chaotropic lysis buffer and highly uniform magnetic silica beads [21].

The V3-V4 hypervariable regions of the bacterial 16S rRNA gene were amplified, according to the 16S metagenomic sequencing library preparation protocol (Illumina, San Diego, CA, USA). Final indexed libraries were prepared by equimolar (4 nmol/L) pooling, denaturation, and dilution to 6 pmol/L before loading onto the MiSeq flow cell (Illumina). A 2 × 300 bp paired-end run was used. Raw reads were analyzed according to a previously described procedure [22]. Briefly, a single fragment from two overlapping pairs was generated using PandaSeq software (v2.5) [23]. Downstream analyses, such as filtering, denoising via the creation of “zero-radius Operational Taxonomic Units” (zOTUs), taxonomy assignments, and diversity analyses were performed using the QIIME suite (release 1.9.0) [24], the unoise3 algorithm [25], the RDP classifier [26], and the SILVA 16S rRNA database (release 132, https://www.arbsilva.de/fileadmin/silva_databases/qiime/Silva_132_release.zip), respectively.

Alpha-diversity evaluation was estimated according to several microbial diversity metrics (i.e., Chao1, Shannon index, Observed Species, Good’s coverage, Faith’s phylogenetic distance). The beta-diversity analysis was conducted using both weighted and unweighted Unifrac distance metrics [27], and through the Principal Coordinates Analysis (PCoA).

## Detection of *Gardnerella vaginalis* clades

Starting from the remaining eluate of the Versant PCR plate, each sample was tested against the four different GV clades with quantitative PCR (qPCR) assays, as previously described [14,28]. The list of primers and probes used are reported in S1 Table.

After the production of a standard curve, a single-plex TaqMan real-time qPCR assay was used for each clade. Briefly, the PCR reaction mixtures (final volume: 25 μL) included 12.5 μL of Platinum Quantitative PCR Supermix-UDG with ROX (Invitrogen, Thermo Fisher, Waltham, MA, USA), 400 nM of each DNA primer, 100 nM of the specific probe, and 2.5 μL of the template. All PCR reactions were performed with the following cycling conditions using a QuantStudio Real-Time PCR system (Applied Biosystems, Thermo Fisher): 45 °C for 3 minutes, 95 °C for 3 minutes, and 40 cycles of 95°C for 15 seconds and 60 °C for 45 seconds. Any reaction which failed to produce a cycling threshold (Ct) value after 40 cycles was recorded as negative. Results were expressed as log_10_ DNA copies/reaction.

## Detection of the putative *G*. *vaginalis* sialidase A gene

The residual DNA of each vaginal sample was used for molecular detection and quantification of the GV sialidase A gene, using the primers and probes described by Lopes dos Santos Santiago *et al*. [29].

Real-time PCR amplifications were performed in reactions (total volume 25 μL) containing 12.5 μL of Platinum Quantitative PCR Supermix-UDG with ROX (Invitrogen), 10 μM of each DNA primer, 5 μM of the specific probe, and 5 μL of the template. All PCR reactions were performed with the following cycling conditions using a QuantStudio Real-Time PCR system (Applied Biosystems): 45 °C for 3 minutes, 95 °C for 10 minutes, and 40 cycles of 95°C for 5 seconds and 58 °C for 10 seconds.

Starting from a quantitative standard curve, the results for each sample were expressed as log_10_ DNA copies/reaction.

### Data analysis and statistics

Statistical analyses were performed by using GraphPad Prism software (version 5.02; GraphPad Software, San Diego, CA, USA). Fisher’s exact test was used to compare categorical data (i.e., presence of GV clades stratified by the vaginal status by Nugent score), whereas one-way analysis of variance (ANOVA) test, followed by Tukey’s multiple comparisons test was used to compare GV or sialidase loads among the different categories. Statistical significance was determined at a p-value < 0.05.

Statistical evaluation of the alpha-diversity indices was performed by non-parametric Monte Carlo-based tests, whereas beta-diversity differences were assessed by a permutation test with pseudo F-ratios (“adonis” function from R package “vegan”, version 2.0–10 [30]. Pairwise relative abundance analysis was performed using a non-parametric Mann–Whitney U test, with Benjamini-Hochberg false-discovery-rate (FDR) correction. For comparing relative abundances across multiple categories, we applied a Kruskal-Wallis test, followed by Dunn’s post hoc test for pairwise comparisons.

Correlation between microbial composition at the genus level and presence/absence of each GV clade was calculated using the point biserial correlation [31], whereas the correlation between microbial profiles and sialidase A quantity was performed using Spearman’s rank-based correlation coefficient on the log_2_-transformed sialidase copy number. Only coefficients showing a p-value of the linear model <0.05 were considered. Statistical evaluations were performed in Matlab (Software version 7.7.0, Natick, MA, USA).

## Results

### Study population

A total of 61 Caucasian pregnant women with a median age of 31.0 years (min-max: 21–44) were enrolled, therefore 122 vaginal samples (61 at the first and 61 at the third trimester) were available for the analyses.

Going from the first to the third trimester of pregnancy, we noticed a significant decrease of cases of BV, together with an increase of cases characterized by a normal microbiota (p<0.001). At the first trimester, 31 (50.8%) women showed a lactobacilli-dominated vaginal flora (Nugent score: 0–3), 19 (31.1%) were characterized by an intermediate microbiota (Nugent score; 4–6), whereas the remaining 11 (18.1%) harbored a BV-associated microbial composition (Nugent score; 7–10). Conversely, at the third trimester, most women (54; 88.5%) were characterized by a normal microbiota, with only 4 cases of BV (6.6%).

When looking to paired vaginal samples from the same women, we found that all the subjects with a normal microbiota at the first trimester (n = 31) maintained the same status at the third trimester, except in three cases where an intermediate microbiota was detected at the end of the pregnancy. All the women with a BV condition at the first trimester (n = 11) showed a normal microbiota at the third trimester, except for 2 cases who continued to have BV-like status by Nugent score. Only two new cases of BV, not present at the beginning of the pregnancy, was observed at the third trimester.

Overall, 85 cases of normal vaginal flora (Health -H- group), 15 BV, and 22 cases of intermediate microbiota (I group) were considered for the analysis.

### Detection and quantification of GV clades

DNA of at least one GV clade was detected in all the vaginal swabs except in four samples (4/122, 3.2%). Two of them belonged to the same woman, negative both at the first (characterized by an intermediate flora) and third trimester (BV condition) of pregnancy. The remaining two cases belonged to two women at the first trimester, with a normal vaginal flora by Nugent score.

Of the 118 positive vaginal swabs, 23 samples were positive for only one clade (18.9%), 47 for two clades (38.5%), and 40 for three clades (32.8%). Eight samples showed the contemporary presence of all four clades (6.6%).

The most common GV clade was represented by clade 4 (101/122; 82.8%), followed by clade 1 (88/122; 72.1%), clade 2 (51/122; 41.8%), and clade 3 (19/122; 23.8%). Detailed results are shown in S1 File. Overall, the distribution of GV clades in the first trimester of pregnancy was similar to that of the third trimester (i.e., almost same number of cases in the two trimesters stratified for GV clade; see S1 File).

Considering the distribution of GV clades stratified by the vaginal status (i.e., Nugent score: H vs I vs BV), a significant association between BV status and clade 2 was found (p = 0.01) (S2 Table).

Interestingly, multiple GV clades (i.e., more than two different clades at the same time) were more often detected in BV conditions than in intermediate or healthy vaginal microbiota (p = 0.035) (S3 Table).

In terms of GV loads (expressed as log_10_ GV copies/reaction ± SD), clade 4 showed the highest mean levels (1.6 ± 1.9), followed by clade 1 (1.5 ± 1.9), clade 3 (1.0 ± 1.9) and clade 2 (0.9 ± 1.5). As shown in Table 1, significantly higher GV loads were found in BV cases compared to healthy conditions, especially for clades 1, 3, and 4. No differences in bacterial loads for any of the clades were found between H and I groups.

**Table 1:** GV bacterial loads stratified by the vaginal status (Nugent score).

Clade	H	I	BV	H vs BV	I vs BV	H vs I
**1**	1.2 ± 1.6	0.6 ± 1.3	3.9 ± 1.9	< 0.0001	< 0.0001	0.37
**2**	0.8 ± 1.5	0.3 ± 0.8	1.9 ± 1.1	0.06	0.08	0.78
**3**	0.5 ± 0.9	0.6 ± 0.3	3.0 ± 2.4	0.007	0.06	0.99
**4**	1.3 ± 0.7	1.4 ± 1.0	3.8 ± 1.3	0.0001	0.003	0.96

GV loads are expressed as log_10_ DNA copies/reaction. Results are expressed as mean ± standard deviation. Statistical significance was deemed for p < 0.05. H = healthy (Nugent Score: 0–3); I = intermediate flora (Nugent Score: 4–6); BV = bacterial vaginosis (Nugent Score: 7–10).

Considering paired vaginal samples, we found that women who cleared their BV status at the third trimester of pregnancy were characterized by a significant reduction of GV clade 1 (4.1 ± 1.9 vs 2.5 ± 2.0; p = 0.01) and clade 4 loads (4.3 ± 1.9 vs 2.7 ± 2.0; p = 0.001). Moreover, in this group, the positivity for clade 2 decreased from 77.7% (7/9) to 22.2% (2/9). The two cases of BV observed at the third trimester, not present at the beginning of the pregnancy, showed a pattern of GV clades/loads similar to the third trimester.

### Presence of the putative GV sialidase A gene

GV sialidase A gene was detected in 89.3% of the samples (109/122). All the negative samples belonged to H (n = 10) or I women (n = 3). When considering quantitative results (expressed as log_10_ DNA copies/reaction), we found higher sialidase levels in BV group (3.9 ± 2.4) compared to both healthy (1.3 ± 1.7; p<0.0001) and intermediate conditions (1.1 ± 1.1; p<0.0001). No significant difference was found between H and I groups (p = 0.9).

Overall, considering paired vaginal samples from the same women, we noticed a significant reduction of sialidase gene count going from the first to the third trimester of pregnancy (2.0 ± 2.2 vs 1.5 ± 1.8, p = 0.02). These data reflected the increase of cases characterized by a normal microbiota at the end of the pregnancy. Women who cleared their BV status at the third trimester of pregnancy showed a significant decrease of sialidase levels (4.3 ± 1.9 vs 2.6 ± 1.3; p = 0.04). The two cases of BV observed at the third trimester, not present at the beginning of the pregnancy were characterized by low levels of sialidase (0.09 ± 0.09).

### Correlation between GV clades and vaginal microbiome

A correlation between the presence of the different GV clades and the vaginal microbiome profiles was performed on the whole dataset (122 vaginal samples), not dividing the two different sampling timepoint (first vs third trimester of pregnancy).

Alpha-diversity evaluation showed a significant (p<0.002) difference in the biodiversity of samples positive or negative for GV clade 2 for all the metrics (i.e., Chao1, Observed Species, Faith’s Phylogenetic tree, Shannon index, Good’s coverage), and for clade 1 (p<0.035, Observed Species and Good’s coverage only), with an increase in biodiversity in positive samples. Moreover, we detected a somewhat significant difference in biodiversity according to the number of clades contemporary found in the vaginal ecosystem, with increased diversity for samples positive for 3 clades compared to samples positive for 1 or 2 clades (p<0.02 for all metrics; S1 Fig).

**Fig 1:**
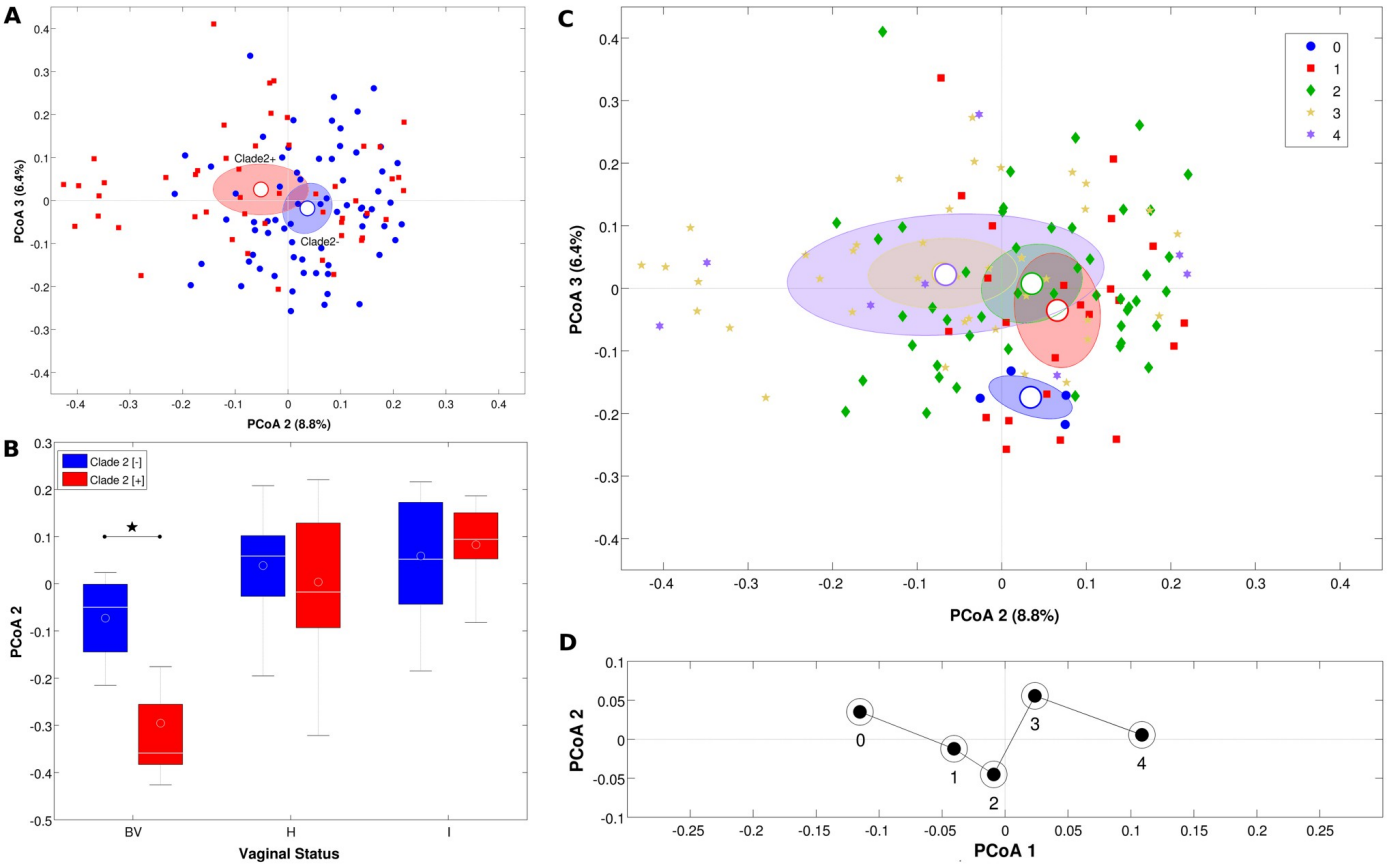
**A**) **Principal Coordinate Analysis (PCoA) plots based on the unweighted Unifrac distance among samples clustered on the presence of GV clade 2**. Each point represents a sample, colored according to the experimental category (blue: Negative, red: Positive). Ellipses are 95% SEM-based confidence intervals, and centroids represent the average coordinate per category. The second and the third coordinates are represented. **B) Boxplots of the distributions of PCoA coordinate 2 on the unweighted Unifrac distances for presence/absence of GV clade 2, with samples divided according to their vaginal status (by Nugent score)**. Asterisk indicates statistical significance (p<0.05, adonis test) **C) PCoA plot based on the unweighted Unifrac distance among samples clustered according to the number of GV clades present in each sample**. Each point represents a sample, which is colored according to the experimental category. Ellipses are 95% SEM-based confidence intervals, and centroids represent the average coordinate per each category. The second and the third coordinates are represented. **D) Trajectory plot of the PCoA centroids obtained from the first and second coordinate of the PCoA analysis of the weighted Unifrac distances**.

The evaluation of the microbial composition of the samples (beta-diversity; Fig 1A) confirmed that the most evident differences were associated with clade 2 (both unweighted and weighted Unifrac distances: p = 0.001 and p = 0.002, respectively). On the other hand, no significant difference in the microbial composition was noticed between positive and negative samples for clades 1, 3, and 4 (with the only exception of weighted Unifrac distance for clade 1, p = 0.044) (S2 Fig).

Stratification of the samples according to the vaginal status (by Nugent score) highlighted the peculiar behavior of the BV samples for clade 2, the only combination that presented a statistically significant difference (p = 0.003) (Fig 1B).

Considering the number of clades simultaneously detected in the same sample, the unweighted Unifrac distances depicted a certain separation between samples negative to all clades and the other samples (although non-significant). On the other hand, samples positive to 3 clades were significantly different (p<0.03, unweighted Unifrac) to those positive to none, 1 or 2 clades; no differences were found between samples with 3 or 4 positive clades (Fig 1C). Moreover, the plot of the weighted Unifrac centroids for each group seemed to depict a sort of trajectory along PCoA first component axis (Fig 1D).

The analysis of bacterial relative abundances at genus level provided further evidence that GV clade 2 could be the one associated with the major shifts in the microbial composition. Major components of the vaginal microbiota were found altered in samples positive for this clade, compared to clade 2-negative samples: *Lactobacillus* was observed to be decreased (-7.3% in clade 2-positive samples), while *Prevotella*, *Megasphaera*, *Sneathia*, and *Dialister* were all increased in relative abundance. In particular, *Megasphaera* and *Sneathia* both had a 14-fold increase, and *Prevotella* in samples positive for clade 2 was 8-times higher than in negative ones. On the other hand, clade 1 showed only an alteration of *Prevotella* (avg. rel. ab: 0.7% vs. <0.1% in clade positive and negative, respectively) plus some minor components, such as *Fastidiosipila* and *Parvimonas* (rel. ab. <0.2%). Detailed results are shown in Table 2.

**Table 2:** Average relative abundances for samples positive and negative to each of the four GV clades.

Clade	Num [+]	Num [–]	Genus	Avg. clade [+] (%)	Avg. clade [-] (%)	p-value	Significance
Clade 1	88	34	*Prevotella 6*	0.65	0.01	0.0053	**
			*DNF00809*	0.19	0.00	0.0187	*
			*Fastidiosipila*	0.17	0.00	0.0143	*
			*Parvimonas*	0.09	0.00	0.0143	*
Clade 2	51	71	*Lactobacillus*	73.07	80.34	0.0191	*
			*Prevotella*	3.92	0.48	0.0189	*
			*Megasphaera*	3.10	0.21	0.0350	*
			*Sneathia*	1.74	0.12	0.0030	**
			*Ureaplasma*	0.33	0.45	0.0419	*
			*Dialister*	0.47	0.15	0.0244	*
			*DNF00809*	0.32	0.00	0.0049	**
			*Fastidiosipila*	0.28	0.00	0.0022	**
			*Peptoniphilus*	0.09	0.10	0.0008	***
			*Dietzia*	0.07	0.11	0.0463	*
			*Porphyromonas*	0.16	0.03	0.0223	*
			*Parvimonas*	0.16	0.00	0.0021	**
Clade 3	29	93	--	--	--	--	--
Clade 4	101	21	--	--	--	--	--

Number of samples per group, as well as the p-value of the Mann-Whitney U-test is reported. Significance was assessed using a Benjamini-Hochberg FDR correction on the 25 most abundant genera (using an FDR of 0.15 as a threshold). For better clarity, in the table, raw unadjusted p-values were provided. Asterisks indicate graphically the statistical significance:

***: p<0.001,

**: p<0.01;

*: p<0.05.

**Fig 2:**
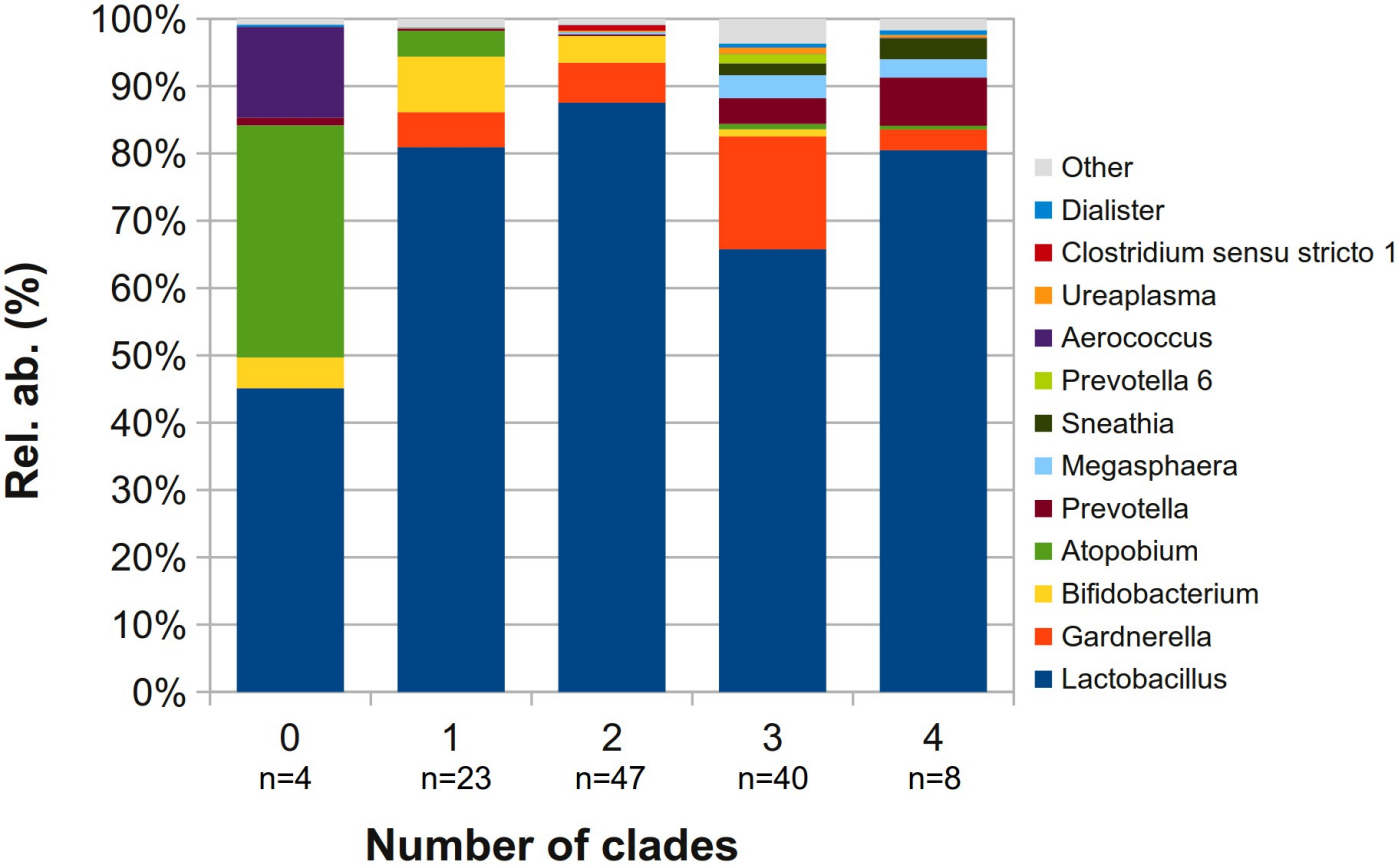
Barplot of the average relative abundance of the main genera, over the number of clades found at the same time in each sample. Only the first 12 most abundant genera are plotted, whereas the remaining genera are grouped in the ‘Others’ category.

When evaluating relative abundances according to the number of GV clades found simultaneously in the same sample, interesting trends were found (Fig 2). In particular, *Bifidobacterium* tended to decrease with the increasing number of positive clades (R = -0.994, Pearson’s correlation coefficient, considering only samples positive to 1, 2 or 3 clades), whereas a group of BV-related bacteria (i.e., *Prevotella*, *Megasphaera*, *Sneathia*, *Prevotella 6*, *Ureaplasma*, and *Dialister*) tended to increase (Pearson’s coefficients between 0.833 and 0.916). No significant trend was found between *Lactobacillus* spp. abundance and GV multi-clade colonization. In vaginal samples negative for GV (n = 4), a significant proportion of *Atopobium* was found (average relative abundance about 30%).

**Fig 3:**
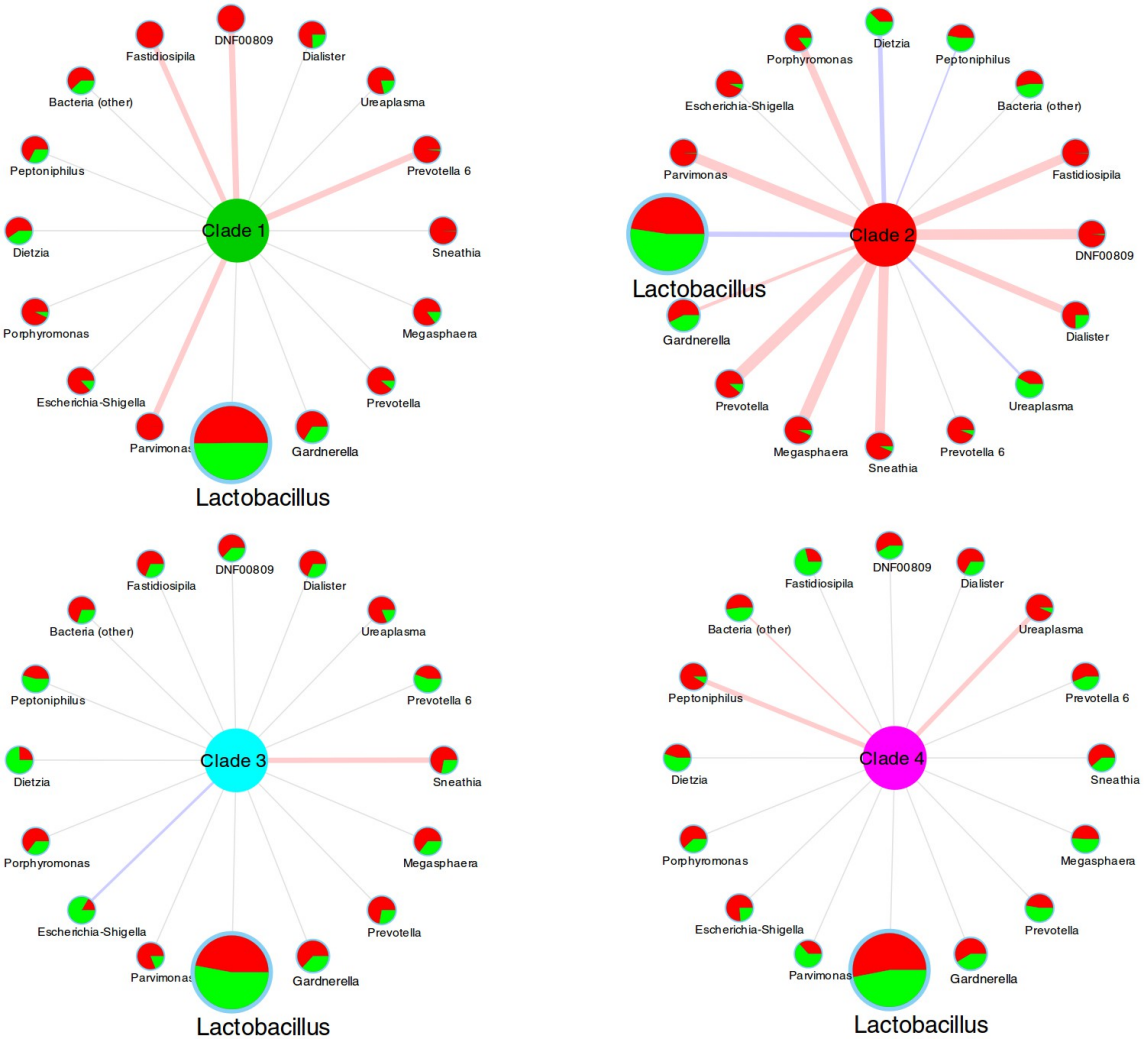
Diagram representing the correlations between bacterial genera and presence/absence of each of the four GV clades. Edge color represents the sign of the correlation (blue = negative, red = positive, gray = not significant) and edge thickness represents the strength of correlation. Node and label size of the bacterial genera is proportional to the average abundance over the whole set of samples. Piecharts in each node represent the skewness of the average relative abundance of the genera in samples not showing (green) or showing (red) the specific clade. Thus, for example, genera with a piechart nearly completely red are those whose abundance in the samples is more associated with positivity for the specific clade. Data used for building the figure are provided in S4 Table.

The alterations suggested by analyzing the relative abundance of bacterial genera were confirmed with the point-biserial correlation between genus-level relative abundances and the presence/absence of each of the four GV clades (Fig 3). Clade 2 was the one displaying most of the correlations: indeed, 13 genera among the first 16 most abundant in the vaginal ecosystem showed a significant correlation. For example, clade 2 was negatively correlated with *Lactobacillus* (R = -0.122) and positively with several BV-associated bacteria (i.e., *Prevotella*, *Megasphaera*, *Sneathia*, *Dialister*, *Coriobacteriales* DNF00809, *Fastidiosipila*, with coefficients in the range 0.201–0.324). On the other hand, all the other GV clades displayed fewer and weaker correlations (i.e.: all significant correlations had R < 0.159, Fig 3). Among them, we observed a positive correlation between GV clade 1 and *Parvimomas*, *Prevotella*, *Coriobacteriales* DNF00809, and *Fastidiosipila*, as well as a positive correlation between clade 4 and *Peptoniphilus* and *Ureaplasma*. Notably, all the significant correlations corresponded to the taxa found as differentially abundant in samples positive or negative to each clade.

### Correlation between sialidase gene levels and vaginal microbiome

A correlation between sialidase gene levels and the vaginal microbiome profiles was performed on the whole dataset (122 vaginal samples).

**Fig 4:**
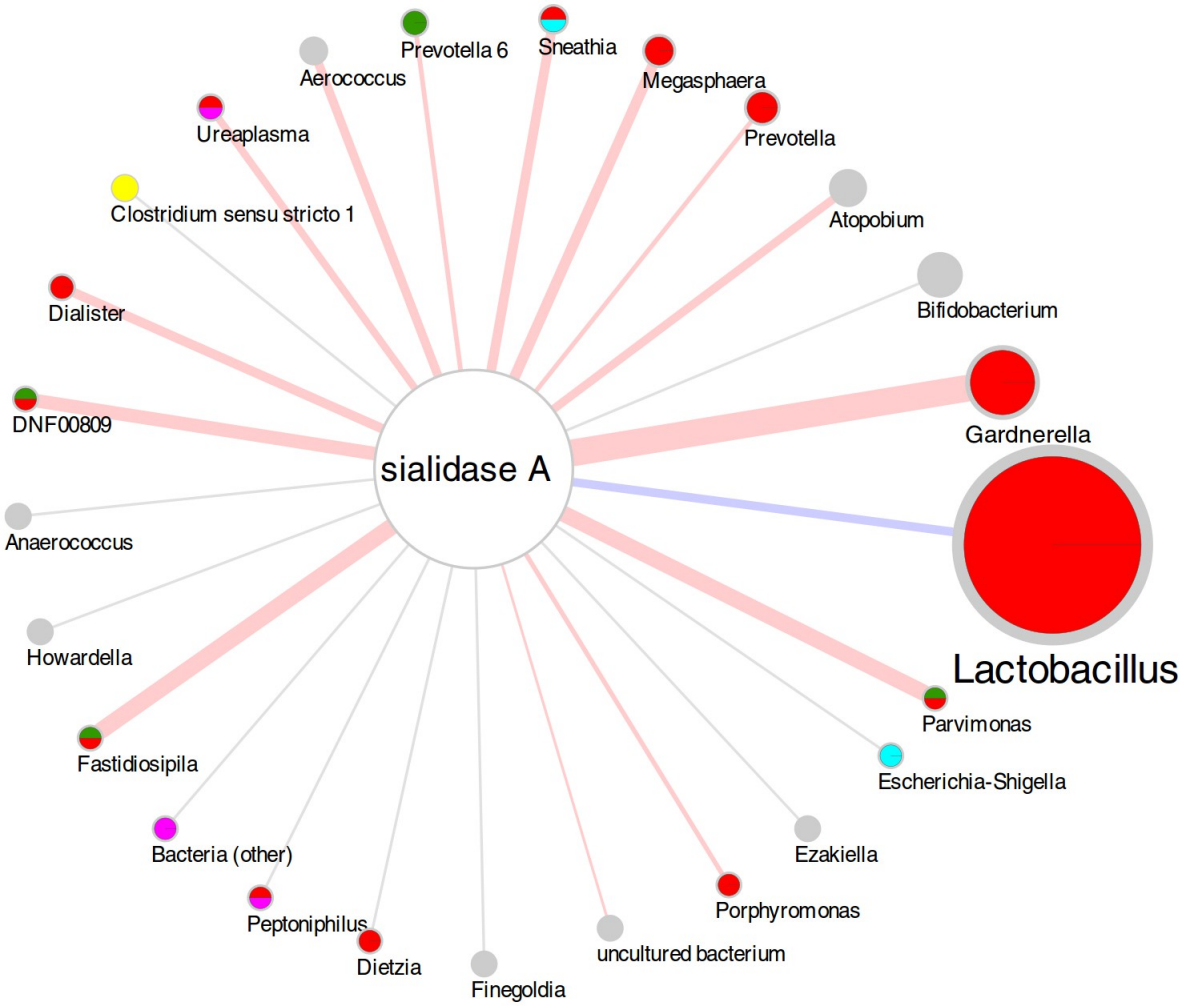
Diagram representing the correlations between bacterial genera and the number of copies of the sialidase gene, on a log_2_ scale. Edge color represents the sign of the correlation (blue = negative, red = positive, gray = not significant) and edge thickness represents the strength of correlation. Node and label size of the bacterial genera is proportional to the average abundance over the whole set of samples. Piecharts in each node indicate whether the genera was differentially abundant in samples positive or negative for each GV clade (green = clade 1; red = clade 2; cyan = clade 3; magenta = clade 4). Colors are the same as the clades in Fig 3. Data used for building the figure are provided in S5 Table.

When evaluating Spearman’s correlation between the sialidase A gene count and the relative abundance of the main bacterial genera (Fig 4), we observed a negative correlation with *Lactobacillus* (R = -0.27) while positive correlations were observed with *Gardnerella* (R = 0.52), *Atopobium* (R = 0.26), *Prevotella* (R = 0.21), *Megasphaera* (R = 0.30), *Sneathia* (R = 0.28), and *Prevotella 6* (R = 0.21). Many of them were also the bacterial genera found as differentially abundant in the comparison between samples positive or negative for each clade.

## Discussion

In this work we explored the distribution and loads of the different GV clades in the vaginal environment of a cohort of Caucasian pregnant women, deciphering the correlations of the different clades with the inhabitants of the vaginal microbiome. Moreover, considering the key role of putative GV sialidase A in the ability to form biofilm and BV pathogenesis, we assessed sialidase gene count in the vaginal ecosystem of each woman [32].

At first, in agreement with previous observations, we found that GV is extremely common even in women harboring a lactobacilli-dominated vaginal microbiome, thus confirming its commensal role in many healthy women [6].

Moreover, the distribution of GV clades found in our population was in line with previously published literature, with clade 4 being the most common one, followed by clade 1, clade 2, and clade 3 [12,14]. When analyzing the distribution of GV stratified by the vaginal status (i.e., Nugent score), we found a significant association between clade 2 and BV condition. In this context, it should be noted that the correlation between single GV clade and BV status is still under debate. Even though most studies indicate clade 1 as the one most associated with BV, even clades 2 and 3 were found related to an abnormal vaginal microbiota, in conjunction with higher Nugent scores [11–14]. In our dataset, the relationship between BV status and GV clade 2 was strengthened by the association of this clade to a higher vaginal microbial biodiversity, as well as to several BV-associated bacteria. Indeed, women positive for clade 2 were more likely to harbor a plethora of anaerobic bacteria in the vaginal ecosystem, typically found during dysbiotic conditions (e.g., *Gardnerella*, *Prevotella*, *Megasphaera*, *Sneathia*, *Dialister*).

Although some studies found a relationship between clade 4 and dysbiotic conditions [13,33], we did not observe this association, being clade 4 very frequently detected also in women with low Nugent scores (i.e., normal lactobacilli-dominated flora). Our results, on the other hand, agree with several other observations suggesting that clade 4 is not associated with BV or altered vaginal microbiota [11,12,14], presumably for its reduced pathogenicity. Indeed clade 4 strains are characterized by low or no production of virulence factors, including sialidase A enzyme, resulting in a reduced ability to mucin degradation and to form biofilm [34,35]. The discrepancies between studies in defining the specific GV clades associated with BV or healthy vaginal status could be due to population differences, including ethnicity, sexual activities and networks, pregnancy status, and behavioral practices such as smoking, diet, and hygiene.

Other interesting data emerged when considering the contemporary presence of more than one GV clade. Indeed, in our study, according to previous observations, BV-women were more likely (p = 0.003) to have multiple clades of GV (i.e., co-colonization with more than two different GV clades) compared to single or dual clades [6,33]. As previously hypothesized, multiple clades may act synergistically to suppress *Lactobacillus* spp. or form biofilms [12]. During BV, biofilm can incorporate various bacteria, including different GV strains/clades, over time, so it can be hypothesized that in BV status, GV is commonly found as multi-clade, in contrast with healthy women characterized by dispersed forms of the microorganism (i.e., single-clade).

In line with these findings, as the number of GV clades increased, we observed an increase in the severity (i.e., higher microbial diversity with an abundance of BV-associated taxa) of vaginal dysbiosis. Indeed, women co-colonized by multi-clade of GV were characterized by lower levels of *Bifidobacterium*, and higher levels of BV-related bacteria, such as *Prevotella*, *Megasphaera*, *Sneathia*, *Ureaplasma*, and *Dialister*.

In this context, it should be remembered that, despite being typically associated with the gut microbiota, some *Bifidobacterium* are frequent and abundant colonizers of the ‘healthy’ vaginal microbiota [36]. Bifidobacteria are able to produce L-lactic acid, which may contribute to lowering vaginal pH values, thus inhibiting the growth of dysbiosis-associated bacteria [36].

Overall, no significant trend was found between lactobacilli abundance and GV multi-clade colonization. However, additional studies based on more in-depth 16s rRNA gene analyses might possibly reveal associations between GV multi-clade colonization and specific *Lactobacillus* species (e.g., *L*. *crispatus*, *L*. *iners*).

During dysbiotic conditions, *Gardnerella* spp. can adhere to glycan-binding sites, and biofilm-forming *Gardnerella* spp. can, then, act as a scaffold for the attachment of other BV-associated species, such as *Atopobium vaginae* and *Prevotella* spp. [32]. We had the chance to find a few cases characterized by a high proportion of *Atopobium* spp. in absence of GV. Even though *Atopobium vaginae* is considered a highly specific marker for BV, especially when combined with GV [37], it should be remembered that this microorganism can also be found in the vaginal microbiota of healthy women and detected without GV [38]. Considering the low number of data points (n = 4), the exact role of *Atopobium* in GV-negative samples remains to be further elucidated.

We found that BV conditions were characterized by higher GV bacterial loads for all the four clades, in agreement with previous observations demonstrating that the quantification of GV clades is a good and accurate predictor of an abnormal vaginal microbiome, associated with BV condition [6].

Similarly, sialidase A gene levels were significantly higher in women with a BV status compared to H and I groups. In agreement with this observation, sialidase gene count was positively correlated with several BV-associated bacteria, such as *Gardnerella*, *Atopobium*, *Prevotella*, *Megasphaera*, and *Sneathia*, and negatively with *Lactobacillus* genera.

Recently, Ferreira *et al*. observed that many BV-related taxa (especially belonging to *Prevotella* genera) were enriched in sialidase-positive vaginal samples and that only two taxa, including *Lactobacillus helveticus*, were associated with sialidase-negative samples [7].

The negative correlation between sialidase gene levels and *Lactobacillus* is not surprising if we consider that these microorganisms are the hallmark of vaginal health and eubiosis, being detected only in low abundances in BV/dysbiotic bacterial communities [2,39].

Considering that sialidase A gene is not the best marker of functional sialidase in vivo [32,34], further studies assessing the presence of additional GV sialidase genes (namely, NanH2 and NanH3) are needed to better understand the correlation between sialidase gene count and BV presence/severity. Indeed, NanH2 and NanH3 enzymes have been recently recognized as the primary sources of sialidase activity in GV [40].

Taken together, our data highlight that BV status is associated with the presence of clade 2, the contemporary presence of multi-clades of GV (i.e., >2 different clades), higher GV bacterial loads, and higher sialidase gene count.

We are fully aware of some limitations of this study. First, the limited number of BV-affected women may have led to missing significant associations between specific GV clade and loads, sialidase gene count, and microbial signatures during vaginal dysbiosis. Second, the use of culture-based approaches will be needed to better understand the role of sialidase enzymes in BV pathogenesis and severity. Finally, detailed information about the clinical manifestations of BV, as well as an evaluation of Amsel criteria of each woman will help to better define the role of GV clades and sialidase activity in the conditions of vaginal dysbiosis.

In conclusion, we added knowledge on the distribution of GV clades in the vaginal ecosystem of a cohort of pregnant women, assessing the potential role of specific GV clades, bacterial loads, and sialidase A gene count in BV pathogenesis. We found specific microbial fingerprints related to the different GV clades, thus underlining the ability of each clade to interact and cooperate in a different way with the inhabitants of the vaginal environment.

Further studies will be needed to understand if GV clades have a different clinical and prognostic impact on pregnancy status, as well as if one or more combined parameters (e.g., number of GV clades, type of clades, GV loads) can predict the severity of BV in pregnant women.

Since a vaginal microbiome enriched in BV-related taxa, is associated with adverse pregnancy outcomes [41,42], a detailed characterization of BV conditions in pregnant women could open new perspectives for the prevention of pregnancy-related complications, such as first-trimester miscarriage and preterm birth.

## Supporting information

S1 Fig Boxplots of alpha-diversity values for samples positive/negative for each of the four GV clades (‘Clade 1, Clade 2, Clade 3, Clade 4’) (A-D), and according to the number of positive clades found contemporary in the same sample (‘Number of clades’) (E). Observed species metric is represented for the single clades, whereas PD whole tree metric is used for ‘number of clades’. Asterisk represents statistical significance of the difference (p<0.05, permutation-based non-parametric t-test).
(TIFF)

S2 Fig Principal Coordinate Analysis (PCoA) plots based on the unweighted Unifrac distance among samples clustered on the presence of GV clades 1(A), 3 (B) and 4 (C). Each point represents a sample, colored according to the experimental category (blue: Negative, red: Positive). Ellipses are 95% SEM-based confidence intervals, and centroids represent the average coordinate per each category. The second and the third coordinates are represented for all plots.
(TIFF)

S1 Table List of primers and probes used for the detection of GV clades. (DOCX)

S2 Table Distribution of GV clades, stratified by the vaginal status (H vs I vs BV, by the Nugent score). (DOCX)

S3 Table Presence of GV clades (single clade, dual clade, multi-clade) stratified by vaginal status (H vs I vs BV, by the Nugent score). (DOCX)

S4 Table Correlation between GV clades presence and bacterial genera. The table reports the data that were used for building Fig 3 in the main paper. For each of the 16 most abundant genera having at least 1 significant correlation (point biserial correlation, p<0.05), the correlation coefficient, as well as the average relative abundance of the bacterial genera for samples positive (“[+]”) or negative (“[-]”) for the presence of the specific GV clade are reported.
(DOCX)

S5 Table Correlation between sialidase and bacterial genera. The table reports the data that were used for building Fig 4 in the main paper. For each of the 25 most abundant genera, the correlation coefficient (point biserial correlation, p<0.05), as well as the average relative abundance of the bacterial genera and the list of clades reporting that genus as significantly altered in the comparison between clade [+] and clade [-] samples are reported.
(DOCX)

S1 File Detailed data about the women enrolled: Vaginal status and GV clade distribution stratified by the different gestational stages. N = negative; P = positive.
(XLSX)

## References

[1] Smith SB, Ravel J. The vaginal microbiota, host defense and reproductive physiology. J Physiol. 2017;595:451–463. doi: 10.1113/JP271694 27373840 PMC5233653

[2] Ceccarani C, Foschi C, Parolin C, D’Antuono A, Gaspari V, Consolandi C, et al. Diversity of vaginal microbiome and metabolome during genital infections. Sci Rep. 2019;9:14095. doi: 10.1038/s41598-019-50410-x 31575935 PMC6773718

[3] Parolin C, Foschi C, Laghi L, Zhu C, Banzola N, Gaspari V, et al. Insights Into Vaginal Bacterial Communities and Metabolic Profiles of *Chlamydia trachomatis* Infection: Positioning Between Eubiosis and Dysbiosis. Front Microbiol. 2018;9:600. doi: 10.3389/fmicb.2018.00600 29643849 PMC5883401

[4] Sobel JD. Bacterial vaginosis. Annu Rev Med. 2000;51:349–56. doi: 10.1146/annurev.med.51.1.349 10774469

[5] Srinivasan S, Morgan MT, Fiedler TL, Djukovic D, Hoffman NG, Raftery D, et al. Metabolic signatures of bacterial vaginosis. mBio 2015;6:e00204–15. doi: 10.1128/mBio.00204-15 25873373 PMC4453549

[6] Shipitsyna E, Krysanova A, Khayrullina G, Shalepo K, Savicheva A, Guschin A, et al. Quantitation of all Four Gardnerella vaginalis Clades Detects Abnormal Vaginal Microbiota Characteristic of Bacterial Vaginosis More Accurately than Putative G. vaginalis Sialidase A Gene Count. Mol Diagn Ther. 2019;23:139–147. doi: 10.1007/s40291-019-00382-5 30721449 PMC6394432

[7] Ferreira CST, Marconi C, Parada CMGL, Ravel J, da Silva MG. Sialidase Activity in the Cervicovaginal Fluid Is Associated With Changes in Bacterial Components of *Lactobacillus*-Deprived Microbiota. Front Cell Infect Microbiol. 2022;11:813520. doi: 10.3389/fcimb.2021.813520 35096658 PMC8793624

[8] Gelber SE, Aguilar JL, Lewis KL, Ratner AJ. Functional and phylogenetic characterization of Vaginolysin, the human-specific cytolysin from Gardnerella vaginalis. J Bacteriol. 2008;190:3896–903. doi: 10.1128/JB.01965-07 18390664 PMC2395025

[9] Swidsinski A, Mendling W, Loening-Baucke V, Ladhoff A, Swidsinski S, Hale LP, et al. Adherent biofilms in bacterial vaginosis. Obstet Gynecol. 2005;106:1013–23. doi: 10.1097/01.AOG.0000183594.45524.d2 16260520

[10] Ahmed A, Earl J, Retchless A, Hillier SL, Rabe LK, Cherpes TL, et al. Comparative genomic analyses of 17 clinical isolates of *Gardnerella vaginalis* provide evidence of multiple genetically isolated clades consistent with subspeciation into genovars. J Bacteriol. 2012;194:3922–37. doi: 10.1128/JB.00056-12 22609915 PMC3416530

[11] Janulaitiene M, Paliulyte V, Grinceviciene S, Zakareviciene J, Vladisauskiene A, Marcinkute A, et al. Prevalence and distribution of *Gardnerella vaginalis* subgroups in women with and without bacterial vaginosis. BMC Infect Dis. 2017;17:394. doi: 10.1186/s12879-017-2501-y 28583109 PMC5460423

[12] Plummer EL, Vodstrcil LA, Murray GL, Fairley CK, Danielewski JA, Garland SM, et al. *Gardnerella vaginalis* Clade Distribution Is Associated With Behavioral Practices and Nugent Score in Women Who Have Sex With Women. J Infect Dis. 2020;221:454–463. doi: 10.1093/infdis/jiz474 31544206

[13] Hilbert DW, Schuyler JA, Adelson ME, Mordechai E, Sobel JD, Gygax SE. *Gardnerella vaginalis* population dynamics in bacterial vaginosis. Eur J Clin Microbiol Infect Dis. 2017;36:1269–1278. doi: 10.1007/s10096-017-2933-8 28197729

[14] Balashov SV, Mordechai E, Adelson ME, Gygax SE. Identification, quantification and subtyping of *Gardnerella vaginalis* in noncultured clinical vaginal samples by quantitative PCR. J Med Microbiol. 2014;63:162–175. doi: 10.1099/jmm.0.066407-0 24200640

[15] Fox C, Eichelberger K. Maternal microbiome and pregnancy outcomes. Fertil Steril. 2015; 104:1358–1363. doi: 10.1016/j.fertnstert.2015.09.037 26493119

[16] Nelson DB, Rockwell LC, Prioleau MD, Goetzl L. The role of the bacterial microbiota on reproductive and pregnancy health. Anaerobe 2016;42:67–73 doi: 10.1016/j.anaerobe.2016.09.001 27612939

[17] Dingens AS, Fairfortune TS, Reed S, Mitchell C. Bacterial vaginosis and adverse outcomes among full-term infants: a cohort study. BMC Pregnancy Childbirth 2016;16:278. doi: 10.1186/s12884-016-1073-y 27658456 PMC5034665

[18] Donders G, Bellen G, Rezeberga D. Aerobic vaginitis in pregnancy. BJOG 2011;118:1163–70. doi: 10.1111/j.1471-0528.2011.03020.x 21668769

[19] Yano J, Sobel JD, Nyirjesy P, Sobel R, Williams VL, Yu Q, et al. Current patient perspectives of vulvovaginal candidiasis: incidence, symptoms, management and post-treatment outcomes. BMC Womens Health 2019;19:48. doi: 10.1186/s12905-019-0748-8 30925872 PMC6441174

[20] Zozaya-Hinchliffe M, Lillis R, Martin DH, Ferris MJ. Quantitative PCR assessments of bacterial species in women with and without bacterial vaginosis. J Clin Microbiol. 2010;48:1812–9. doi: 10.1128/JCM.00851-09 20305015 PMC2863870

[21] Marangoni A, Foschi C, Nardini P, Compri M, Cevenini R. Evaluation of the Versant CT/GC DNA 1.0 assay (kPCR) for the detection of extra-genital *Chlamydia trachomatis* and *Neisseria gonorrhoeae* infections. PLoS One 2015;10:e0120979. doi: 10.1371/journal.pone.0120979 25799263 PMC4370730

[22] Severgnini M, Camboni T, Ceccarani C, Morselli S, Cantiani A, Zagonari S, et al. Distribution of *ermB*, *ermF*, *tet*(*W*), and *tet*(*M*) Resistance Genes in the Vaginal Ecosystem of Women during Pregnancy and Puerperium. Pathogens 2021;10:1546. doi: 10.3390/pathogens10121546 34959501 PMC8705968

[23] Masella AP, Bartram AK, Truszkowski JM, Brown DG, Neufeld JD. PANDAseq: paired-end assembler for illumina sequences. BMC Bioinformatics 2012;13:31. doi: 10.1186/1471-2105-13-31 22333067 PMC3471323

[24] Caporaso JG, Kuczynski J, Stombaugh J, Bittinger K, Bushman FD, Costello EK, et al. Correspondence QIIME allows analysis of high throughput community sequencing data Intensity normalization improves color calling in SOLiD sequencing. Nat Methods 2010;7:335–336. 20383131 10.1038/nmeth.f.303 PMC3156573

[25] Edgar RC. UNOISE2: Improved error-correction for Illumina 16S and ITS amplicon sequencing. BioRxiv. 2016:081257.

[26] Wang Q, Garrity GM, Tiedje JM, Cole JR. Naive Bayesian classifier for rapid assignment of rRNA sequences into the new bacterial taxonomy. Appl Environ Microbiol. 2007;73:5261–7. doi: 10.1128/AEM.00062-07 17586664 PMC1950982

[27] Lozupone C, Lladser ME, Knights D, Stombaugh J, Knight R. UniFrac: An effective distance metric for microbial community comparison. ISME J. 2011;5:169–172 doi: 10.1038/ismej.2010.133 20827291 PMC3105689

[28] Schuyler JA, Mordechai E, Adelson ME, Sobel JD, Gygax SE, Hilbert DW. Identification of intrinsically metronidazole-resistant clades of *Gardnerella vaginalis*. Diagn Microbiol Infect Dis. 2016;84:1–3 doi: 10.1016/j.diagmicrobio.2015.10.006 26514076

[29] Santiago GL, Deschaght P, El Aila N, Kiama TN, Verstraelen H, Jefferson KK, et al. *Gardnerella vaginalis* comprises three distinct genotypes of which only two produce sialidase. Am J Obstet Gynecol. 2011;204:450.e1–7. doi: 10.1016/j.ajog.2010.12.061 21444061

[30] Oksanen J, Blanchet FG, Kindt R, Legendre, Minchin PR, O’Hara RB, et al. Package “Vegan”. R Package Version 2.0–10. 2013. https://cran.r-project.org/src/contrib/Archive/vegan/vegan_2.0-10.tar.gz (accessed on 22 February 2022).

[31] Gupta SD. Point biserial correlation coefficient and its generalization. Psychometrika 1960;25:393–408.

[32] Hardy L, Jespers V, Van den Bulck M, Buyze J, Mwambarangwe L, Musengamana V, et al. The presence of the putative *Gardnerella vaginalis* sialidase A gene in vaginal specimens is associated with bacterial vaginosis biofilm. PLoS One 2017;12:e0172522. doi: 10.1371/journal.pone.0172522 28241058 PMC5328246

[33] Vodstrcil LA, Twin J, Garland SM, Fairley CK, Hocking JS, Law MG, et al. The influence of sexual activity on the vaginal microbiota and *Gardnerella vaginalis* clade diversity in young women. PLoS One 2017;12:e0171856. doi: 10.1371/journal.pone.0171856 28234976 PMC5325229

[34] Schellenberg JJ, Paramel Jayaprakash T, Withana Gamage N, Patterson MH, et al. *Gardnerella vaginalis* Subgroups Defined by cpn60 Sequencing and Sialidase Activity in Isolates from Canada, Belgium and Kenya. PLoS One 2016;11:e0146510. doi: 10.1371/journal.pone.0146510 26751374 PMC4709144

[35] Janulaitiene M, Gegzna V, Baranauskiene L, Bulavaitė A, Simanavicius M, Pleckaityte M. Phenotypic characterization of *Gardnerella vaginalis* subgroups suggests differences in their virulence potential. PLoS One 2018;13:e0200625. doi: 10.1371/journal.pone.0200625 30001418 PMC6042761

[36] Freitas AC, Hill JE. Quantification, Isolation and Characterization of Bifidobacterium From the Vaginal Microbiomes of Reproductive Aged Women. Anaerobe 2017;47:145–156. doi: 10.1016/j.anaerobe.2017.05.012 28552417

[37] Sousa LGV, Castro J, França A, Almeida C, Muzny CA, Cerca N. A New PNA-FISH Probe Targeting *Fannyhessea vaginae*. Front Cell Infect Microbiol. 2021;11:779376. doi: 10.3389/fcimb.2021.779376 34869078 PMC8637528

[38] Bradshaw CS, Tabrizi SN, Fairley CK, Morton AN, Rudland E, Garland SM. The association of *Atopobium vaginae* and *Gardnerella vaginalis* with bacterial vaginosis and recurrence after oral metronidazole therapy. J Infect Dis. 2006;194(6):828–36. doi: 10.1086/506621 16941351

[39] Marconi C, El-Zein M, Ravel J, Ma B, Lima MD, Carvalho NS, et al. Characterization of the Vaginal Microbiome in Women of Reproductive Age From 5 Regions in Brazil. Sex Transm Dis. 2020;47:562–569. doi: 10.1097/OLQ.0000000000001204 32520883

[40] Robinson LS, Schwebke J, Lewis WG, Lewis AL. Identification and characterization of NanH2 and NanH3, enzymes responsible for sialidase activity in the vaginal bacterium *Gardnerella vaginalis*. J Biol Chem. 2019;294(14):5230–5245. doi: 10.1074/jbc.RA118.006221 30723162 PMC6462536

[41] Dall’Asta M, Laghi L, Morselli S, Re MC, Zagonari S, Patuelli G, et al. Pre-Pregnancy Diet and Vaginal Environment in Caucasian Pregnant Women: An Exploratory Study. Front Mol Biosci. 2021;8:702370. doi: 10.3389/fmolb.2021.702370 34395531 PMC8356051

[42] Di Simone N, Santamaria Ortiz A, Specchia M, Tersigni C, Villa P, et al. Recent Insights on the Maternal Microbiota: Impact on Pregnancy Outcomes. Front Immunol. 2020;11:528202. doi: 10.3389/fimmu.2020.528202 33193302 PMC7645041

