# Supplementary figures and images for: Gardnerella vaginalis clades in pregnancy: New insights into the interactions with the vaginal microbiome
Source: PLoS One. 2022 Jun 14;17(6):e0269590. doi: 10.1371/journal.pone.0269590 (PMC9197028; doi:10.1371/journal.pone.0269590)

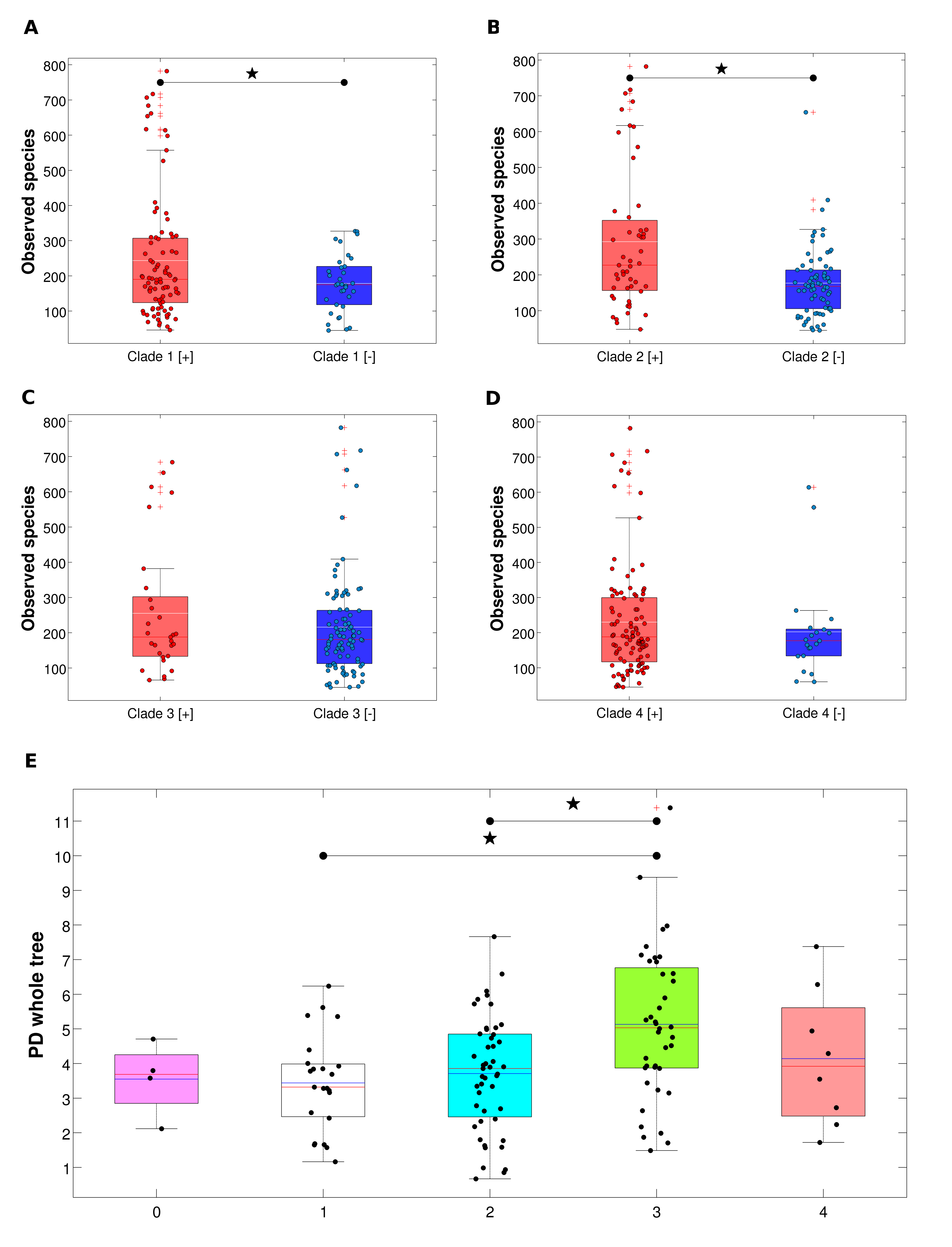

Supplement: S1 Fig — Observed species metric is represented for the single clades, whereas PD whole tree metric is used for ‘number of clades’. Asterisk represents statistical significance of the difference (p<0.05, permutation-based non-parametric t-test). (TIFF) [file pone.0269590.s001.tiff]

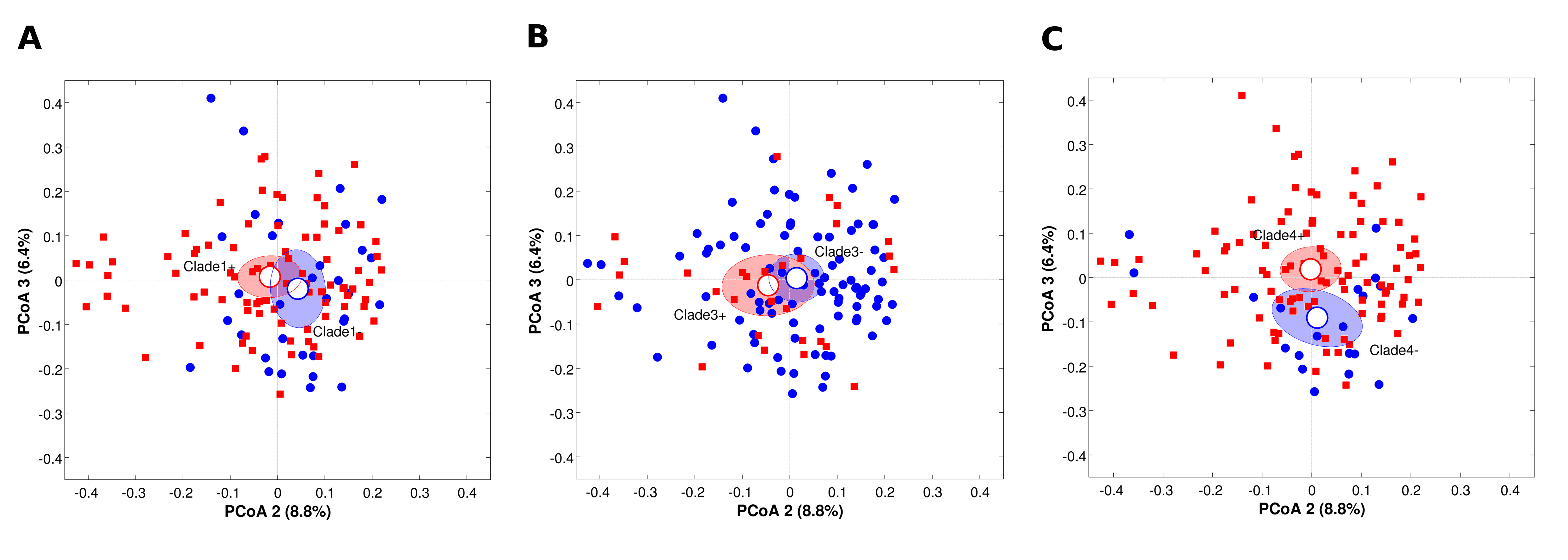

Supplement: S2 Fig — Each point represents a sample, colored according to the experimental category (blue: Negative, red: Positive). Ellipses are 95% SEM-based confidence intervals, and centroids represent the average coordinate per each category. The second and the third coordinates are represented for all plots. (TIFF) [file pone.0269590.s002.tiff]
