# Supplementary material for: Gardnerella vaginalis clades in pregnancy: New insights into the interactions with the vaginal microbiome
Source: PLoS One. 2022 Jun 14;17(6):e0269590. doi: 10.1371/journal.pone.0269590 (PMC9197028; doi:10.1371/journal.pone.0269590)
Supplement: S1 Table — (DOCX) [file pone.0269590.s003.docx]

| **Target** | **Primers/probes** |
| --- | --- |
| **GV clade 1**  (Putative a-L-fucosidase) | 5’-CCAGTCATAAGTTTGCGTTTTACC-3’  5’-TGGCACTGGCAAAGTTTACAAC-3’  5’-FAM-CTCGCCGCAAGCACCATCAAGCCA-3’ |
| **GV clade 2**  (Hypothetical protein) | 5’-GCAAAGCAGACTGAGCGTATTAG-3’  5’-GTAATAATCAGGCTCCTCATCGC-3’  5’-FAM-CGCAGGCGCTCGCATAACAGTGCA-3’ |
| **GV clade 3**  (Thioredoxin) | 5’-TTCTGCTTCTTCTGCTATTTGCTG-3’  5’-TTCGTTGACTTTTGGGCAACATG-3’  5’-FAM-CGGTCCGTGCCGTTCATTTGGTCC-3’ |
| **GV clade 4**  (Chloride transporter, CIC family) | 5’-CCTACGCAAGCTCCAGACGAC-3’  5’-ACAAGTTGCACTCTTCGAGCTGG-3’  5’-FAM-ACTCGGCTGAAGCACACCACCACT-3’ |

**S1 Table.**
