# Supplementary material for: Gardnerella vaginalis clades in pregnancy: New insights into the interactions with the vaginal microbiome
Source: PLoS One. 2022 Jun 14;17(6):e0269590. doi: 10.1371/journal.pone.0269590 (PMC9197028; doi:10.1371/journal.pone.0269590)
Supplement: S2 Table — (DOCX) [file pone.0269590.s004.docx]

**S2 Table.**

|  | **Clade 1** | | **Clade 2** | | **Clade 3** | | **Clade 4** | |
| --- | --- | --- | --- | --- | --- | --- | --- | --- |
|  | **+** | **-** | **+** | **-** | **+** | **-** | **+** | **-** |
| **BV** | 15.9% | 2.9% | 21.6% | 5.6% | 20.7% | 9.7% | 11.9% | 14.3% |
| **H** | 65.9% | 79.4% | 66.7% | 71.8% | 65.5% | 71.0% | 72.3% | 57.1% |
| **I** | 18.2% | 17.7% | 11.8% | 22.5% | 13.8% | 19.4% | 15.8% | 28.6% |
