# Supplementary material for: Gardnerella vaginalis clades in pregnancy: New insights into the interactions with the vaginal microbiome
Source: PLoS One. 2022 Jun 14;17(6):e0269590. doi: 10.1371/journal.pone.0269590 (PMC9197028; doi:10.1371/journal.pone.0269590)
Supplement: S3 Table — (DOCX) [file pone.0269590.s005.docx]

**S3 Table.**

| Number of clades | H (n=83) | I (n=21) | BV (n=14) |
| --- | --- | --- | --- |
| 1 | 21.6% | 23.8% | 0% |
| 2 | 42.3% | 52.4% | 7% |
| >2 | 36.1% | 23.8% | 93% |
