# Supplementary material for: Gardnerella vaginalis clades in pregnancy: New insights into the interactions with the vaginal microbiome
Source: PLoS One. 2022 Jun 14;17(6):e0269590. doi: 10.1371/journal.pone.0269590 (PMC9197028; doi:10.1371/journal.pone.0269590)
Supplement: S4 Table — The table reports the data that were used for building Fig 3 in the main paper. For each of the 16 most abundant genera having at least 1 significant correlation (point biserial correlation, p<0.05), the correlation coefficient, as well as the average relative abundance of the bacterial genera for samples positive (“[+]”) or negative (“[-]”) for the presence of the specific GV clade are reported. (DOCX) [file pone.0269590.s006.docx]

**S4 Table**

|  | **Point biserial correlation** | | | | | **Average relative abundances** | | | | | | | |
| --- | --- | --- | --- | --- | --- | --- | --- | --- | --- | --- | --- | --- | --- |
| **Genus** | **Clade 1** | **Clade 2** | **Clade 3** | **Clade 4** | **Clade 1+** | | **Clade 1-** | **Clade 2+** | **Clade 2-** | **Clade 3+** | **Clade 3-** | **Clade 4+** | **Clade 4-** |
| Lactobacillus | -- | -0.122 | -- | -- | 77.4 | | 76.9 | 73.1 | 80.3 | 70.6 | 79.4 | 78.9 | 69.6 |
| Gardnerella | -- | 0.071 | -- | -- | 10.4 | | 5.3 | 10.6 | 7.8 | 13.2 | 7.7 | 9.4 | 6.7 |
| Prevotella | -- | 0.324 | -- | -- | 2.5 | | 0.3 | 3.9 | 0.5 | 3.6 | 1.4 | 1.9 | 2.1 |
| Megasphaera | -- | 0.305 | -- | -- | 1.8 | | 0.3 | 3.1 | 0.2 | 2.2 | 1.2 | 1.4 | 1.5 |
| Sneathia | -- | 0.262 | 0.127 | -- | 1.1 | | 0.0 | 1.7 | 0.1 | 1.5 | 0.6 | 0.9 | 0.5 |
| Prevotella 6 | 0.159 | -- | -- | -- | 0.6 | | 0.0 | 1.0 | 0.1 | 0.4 | 0.5 | 0.5 | 0.4 |
| Ureaplasma | -- | -0.04 | -- | 0.113 | 0.5 | | 0.1 | 0.3 | 0.5 | 1.0 | 0.2 | 0.5 | 0.0 |
| Dialister | -- | 0.201 | -- | -- | 0.4 | | 0.1 | 0.5 | 0.2 | 0.5 | 0.2 | 0.3 | 0.2 |
| DNF00809 | 0.153 | 0.282 | -- | -- | 0.2 | | 0.0 | 0.3 | 0.0 | 0.2 | 0.1 | 0.1 | 0.1 |
| Fastidiosipila | 0.131 | 0.247 | -- | -- | 0.2 | | 0.0 | 0.3 | 0.0 | 0.2 | 0.1 | 0.1 | 0.2 |
| Bacteria (other) | -- | -- | -- | 0.017 | 0.1 | | 0.1 | 0.1 | 0.1 | 0.2 | 0.1 | 0.1 | 0.1 |
| Peptoniphilus | -- | -0.015 | -- | 0.117 | 0.1 | | 0.1 | 0.1 | 0.1 | 0.1 | 0.1 | 0.1 | 0.0 |
| Dietzia | -- | -0.105 | -- | -- | 0.1 | | 0.1 | 0.1 | 0.1 | 0 | 0.1 | 0.1 | 0.1 |
| Porphyromonas | -- | 0.192 | -- | -- | 0.1 | | 0.0 | 0.2 | 0.0 | 0.1 | 0.1 | 0.1 | 0.1 |
| Escherichia-Shigella | -- | -- | -0.044 | -- | 0.1 | | 0.0 | 0.2 | 0.0 | 0.0 | 0.1 | 0.1 | 0.0 |
| Parvimonas | 0.144 | 0.268 | -- | -- | 0.1 | | 0.0 | 0.2 | 0.0 | 0.2 | 0.0 | 0.1 | 0.1 |
