# Supplementary material for: Gardnerella vaginalis clades in pregnancy: New insights into the interactions with the vaginal microbiome
Source: PLoS One. 2022 Jun 14;17(6):e0269590. doi: 10.1371/journal.pone.0269590 (PMC9197028; doi:10.1371/journal.pone.0269590)
Supplement: S5 Table — The table reports the data that were used for building Fig 4 in the main paper. For each of the 25 most abundant genera, the correlation coefficient (point biserial correlation, p<0.05), as well as the average relative abundance of the bacterial genera and the list of clades reporting that genus as significantly altered in the comparison between clade [+] and clade [-] samples are reported. (DOCX) [file pone.0269590.s007.docx]

**S5 Table**

| **Genus** | **Correlation** | **Avg. rel. ab.** | **Significance** |
| --- | --- | --- | --- |
| Lactobacillus | -0.265 | 77.30 | Clade 2 |
| Gardnerella | 0.522 | 8.97 | Clade 2 |
| Bifidobacterium | -- | 3.57 | -- |
| Atopobium | 0.263 | 2.16 | -- |
| Prevotella | 0.208 | 1.92 | Clade 2 |
| Megasphaera | 0.296 | 1.42 | Clade 2 |
| Sneathia | 0.281 | 0.80 | Clade 2, Clade 3 |
| Prevotella 6 | 0.21 | 0.47 | Clade 1 |
| Aerococcus | 0.269 | 0.46 | -- |
| Ureaplasma | 0.25 | 0.40 | Clade , Clade 4 |
| Clostridium sensu stricto 1 | -- | 0.33 | -- |
| Dialister | 0.278 | 0.28 | Clade 2 |
| DNF00809 | 0.335 | 0.14 | Clade 1, Clade 2 |
| Anaerococcus | -- | 0.14 | -- |
| Howardella | -- | 0.12 | -- |
| Fastidiosipila | 0.379 | 0.12 | Clade 1, Clade 2 |
| Bacteria (other) | -- | 0.12 | Clade 4 |
| Peptoniphilus | -- | 0.10 | Clade 2, Clade 4 |
| Dietzia | -- | 0.09 | Clade 2 |
| Finegoldia | -- | 0.09 | -- |
| uncultured bacterium | 0.185 | 0.08 | -- |
| Porphyromonas | 0.211 | 0.08 | Clade 2 |
| Ezakiella | -- | 0.08 | -- |
| Escherichia-Shigella | -- | 0.08 | Clade 3, |
| Parvimonas | 0.374 | 0.07 | Clade 1, Clade 2 |
